# Attitudes and Beliefs of Eastern European Consumers Towards Animal Welfare

**DOI:** 10.3390/ani10071220

**Published:** 2020-07-17

**Authors:** Igor Tomasevic, Ivan Bahelka, Jaroslav Čítek, Marjeta Čandek-Potokar, Ilija Djekić, Andriy Getya, Luis Guerrero, Sonya Ivanova, Goran Kušec, Dimitar Nakov, Bartosz Sołowiej, Maricica Stoica, Csaba Szabó, Liliana Tudoreanu, Ulrike Weiler, Maria Font-i-Furnols

**Affiliations:** 1 Faculty of Agriculture, University of Belgrade, Nemanjina 6, 11080 Belgrade, Serbia; idjekic@agrif.bg.ac.rs; 2 Czech University of Life Sciences, Kamýcká 129, 165 21 Prague, Czech Republic; bahelka@af.czu.cz; 3 Research Institute for Animal Production, Hlohovecka 2, 951 41 Luzianky, Slovakia; citek@af.czu.cz; 4 Agricultural Institute of Slovenia, Hacquetova ul. 17, 1000 Ljubljana, Slovenia; meta.candek-potokar@kis.si; 5 National University of Life and Environmental Sciences of Ukraine, Heroiv Oborony str., 12, 03041 Kyiv, Ukraine; getya@ukr.net; 6 IRTA-Food Industries, Granja Camps i Armet, E-17121 Monells, Spain; Lluis.Guerrero@irta.cat; 7 Agricultural Academy, 30 Suhodolska str., 1373 Sofia, Bulgaria; ivanovapeneva@yahoo.com; 8 Faculty of Agrobiotechnical Sciences Osijek, Josip Juraj Strossmayer University of Osijek, Vladimira Preloga 1, 31 000 Osijek, Croatia; gkusec@fazos.hr; 9 Faculty of Agricultural Sciences and Food, University Ss.Cyril and Methodius in Skopje, blvd. Aleksandar Makedonski bb, 1000 Skopje, North Macedonia; nakovd@fznh.ukim.edu.mk; 10 Faculty of Food Sciences and Biotechnology, University of Life Sciences in Lublin, Skromna 8, 20-704 Lublin, Poland; bartosz.solowiej@up.lublin.pl; 11 Cross-Border Faculty, Dunarea de Jos University, 47 Domneasca str., 800008 Galati, Romania; maricica.Stoica@ugal.ro; 12 Faculty of Agricultural and Food Sciences and Environmental Management, University of Debrecen, Boszormenyi ut 138., 4032 Debrecen, Hungary; szabo.csaba@agr.unideb.hu; 13 University of Agronomic Sciences and Veterinary Medicine, Bd Marasti 59, 011464 Bucuresti, Romania; liliana_tudoreanu223@hotmail.co.uk; 14 Universitaet Hohenheim, 460 f Garben str. 17/208, 70593 Stuttgart, Germany; ulrike.weiler@uni-hohenheim.de

**Keywords:** cluster, management, ethical, meat quality

## Abstract

**Simple Summary:**

A survey was conducted with 5508 consumers from 13 Eastern European countries. Three clusters of consumers were identified: one with consumers indifferent towards animal welfare; one with consumers concerned about animal welfare, but they believe it is difficult to achieve; and one with consumers concerned about animal welfare, and they believe it is possible to achieve it.

**Abstract:**

The aim of this exploratory work, because of the existing bias on the size of the sample and some of the sociodemographic characteristics of the participants, was to investigate the Eastern European consumers’ beliefs and attitudes toward animal welfare, to perform a cross-country segmentation analysis and to observe possible differences with their Western European counterparts. For this purpose, a survey was conducted with 5508 consumers from 13 Eastern European countries (Bosnia and Herzegovina, Bulgaria, Czech Republic, Croatia, North Macedonia, Hungary, Moldova, Poland, Romania, Serbia, Slovakia, Slovenia, and Ukraine) using a questionnaire with nine statements about consumers beliefs regarding animal welfare (aspects of management, ethical issues about animals, and consequences of animal welfare on meat quality and price), one statement about the willingness to pay more for meat produced under better welfare conditions, and four statements regarding attitudes toward animal welfare. Differences between countries were detected for all the statements. Moreover, three clusters of consumers were identified: one with consumers indifferent towards animal welfare; one with consumers concerned about animal welfare, but they believe it is difficult to achieve; and one with consumers concerned about animal welfare, and they believe it is possible to achieve it.

## 1. Introduction

Public concern for the suffering and well-being of animals used in food system is ever-increasing [1]. The reasons for this concern are related to the respect for animals or to the sense of right doing, human interspecies empathy, and moral values, as well as to the effect of poor welfare on meat and/or product quality on a market share [2]. Consumer’s perceptions, beliefs and attitudes towards animal welfare have been studied in several countries [3,4,5]. At the European Union (EU) level, animal welfare has not only been a topic of discussion for several decades, the respect for welfare of animals as sentient beings is part of its legislative foundation (Treaty) and agricultural, transport, internal market, and research policies. There is a legislative strategy where minimum standards of animal welfare are imposed by formal regulations. The first European rule was from 1974, and it was related to stunning of animals before slaughter [6]. It was followed by a directive regarding the protection of animals during transportation in 1977 [7]. After that, several regulations have been adopted in order to improve the welfare of animals, setting up minimum standards for transport, stunning, and slaughter and trying to fulfill the expectations of citizens and market demands. Ideally, minimum level of animal welfare and harmonization of production conditions across the EU member states will be provided by EU regulations (Table S1).

Increased public concern foresees ethical ideals of animal treatment and levels of animal welfare that are higher than those regulated by rules and regulations. Achieving a good level of animal welfare represents the goal shared by the majority of European consumers, although the extent of such an interest vary considerably between countries [8,9,10]. Almost half of the EU consumers (45%) believe that, in the EU, the animal welfare and protection is better than in other parts of the world [8], while vast majority of them (89%) believe that food products imported from outside of the EU should have similar animal welfare standards [9].

In order to define the consumer perception towards several aspects related with animal welfare, the European Commission has ordered three official surveys, the so-called “Eurobarometers” [8,9,10]. They provided an insight into profound cultural and historical diversities between different EU countries and an outlook over European consumers’ attitudes about animal welfare. Several scientific studies exploring the nature of public concerns over farm animal welfare, including a number of studies investigating consumer’s attitudes, perceptions, and behavior associated with it have been carried out. Most studies conducted in Europe included mainly Northern and Western European countries.

Therefore, the present study had two aims. The first, to investigate the Eastern European consumers’ beliefs and attitudes regarding animal welfare, and the second is to perform a cross-country segmentation analysis of consumers with specific beliefs and attitudes towards animal welfare.

## 2. Materials and Methods

### 2.1. Consumers and Questionnaire

A survey on consumers’ attitudes and believes about animal welfare was conducted during 2017 using a questionnaire directed at 5508 consumers from 13 Eastern European countries (Bosnia and Herzegovina, Bulgaria, Czech Republic, Croatia, North Macedonia, Hungary, Moldova, Poland, Romania, Serbia, Slovakia, Slovenia, and Ukraine). After constructing the English version of the questionnaire, bilingual expert panels (in English and the target language for translation) were asked to translate it into a local language. Expert panels identified and resolved all the inadequate expressions/concepts of the translation before the questionnaire was translated back to English by an independent translator, bilingual native speaker of the source language. The original and back-translated versions were then compared for the accuracy (differences and similarities).

Only completely answered questionnaires were included in the statistical analysis, so a total of 5384 were further processed. Consumers that participated in this study were 18 years or older and we were looking for a minimum of 200 consumers per country. Participants were recruited by means of professional and family acquaintances and their networks. Although we were looking for a general population (meat and non-meat eaters), in a couple of countries (to find a sufficient number of respondents), the interviews were also conducted at butcher shops and meat markets. However, their share in a total number of respondents was small enough not to bias the overall results. Participants were not economically rewarded. 

The structured questionnaire consisted of three sections. The first section included general demographic information about the respondents (Table 1). The second section consisted of nine statements declaring beliefs about animal welfare. These statements comprised beliefs dealing with particular aspects of animal management (transport, rearing space, way of rearing, and slaughter), possible consequences of higher animals’ welfare standards on meat (quality and price) and ethical issues (dignity, mistreatment and welfare). Furthermore, one additional statement was added about their willingness to pay a little more for the meat from animals treated with dignity. Respondents rated their agreement with each statement on a seven-point Likert scale from 1 “disagree very strongly”, 2 “disagree strongly”, 3 “disagree”, 4 “neither agree nor disagree”, 5 “agree”, 6 “agree strongly” to 7 “agree very strongly”. The third section consisted of four statements on consumers’ attitudes towards animal welfare. Seven-point bipolar scales having different adjectives were selected: 1 “harmful”–7 “beneficial”; 1 “easy”–7 “difficult”; 1 “bad”–7 “good”; and 1 “natural”–7 “artificial”. No information was provided to the participants in order to obtain true situation regarding the attitude of the consumers towards animal welfare.

### 2.2. Statistical Processing

Statistical analyses were performed by means of SAS software (SAS v. 9.4, SAS Institute Inc., Cary, NC, USA) and XLSTAT (v.2017, Addinsoft, Paris, FR, UK). GLM (general linear model) procedure of SAS was used, including country, gender, age group, education level, growing place, and economic situation as fixed effects. Multiple comparison of differences between main effects was performed with Tukey test. Due to the non-normal distribution of the answers in some cases, a previous non-parametric Kruskal–Wallis test was performed. Since the non-parametric test provided results similar to those obtained by the GLM procedure, the later was kept as suggested by O’Mahony [11].

An agglomerative hierarchical cluster analysis with Ward method and Euclidean distance was conducted to segment the participants based on their answers. GLM procedure was performed to determine significant differences among clusters, and Tukey test was used for post-hoc comparison.

## 3. Results and Discussion

### 3.1. Demographic Profile

Table 1 shows the demographic profile of the sample of consumers that participated in the survey by country and as a pool. Regarding the gender, there were 55.3% of female responders. Women representation was higher (above 60%) than average in Czech Republic, Croatia, Moldova, Slovakia, and Ukraine. Consumers were relatively evenly spread by age; the group of consumers with less than 36 years old represented 38.2 %; the group of consumers between 36 and 55 years old represented 34.3%; and the group of consumers above 55 years represented 27.5%. Moldova was the country with the highest proportion of young consumers (<36 years old). Only a fraction of consumers (5.4%) had elementary education, while majority of them had a university degree diploma (52.4%). The percentage of consumers with a university degree was higher than 50% in Croatia, Moldova, Poland, Slovenia, and Ukraine because most of the questionnaires in these countries were distributed among students and their families. Most of the consumers (55.5%) grew up in what they consider urban areas, especially in Bulgaria and Ukraine. When asked about their economic situation, a fifth of the consumers (21.7%) described it as difficult, almost two-thirds of the consumers (63.6%) perceived themselves as a middle class, and the rest (14.7%) believed themselves to be wealthy. Middle class included more than 50% of consumers in all the countries, except Bosnia and Herzegovina and Romania. As explained by Tomasevic, et al. [12], the present study should be considered as exploratory approximation because of the existing bias on some of the sociodemographic characteristics of the participants.

### 3.2. Beliefs about Animal Welfare

#### 3.2.1. Beliefs about Particular Management Aspects

According to Eurobarometer, animal welfare is an important issue for EU citizens [9]. Transport and slaughter conditions reducing suffering are aspects important for consumers [13]. Citizens also consider that both, transport conditions, and animal slaughter, are under the umbrella of European legislation [8]. Nevertheless, it seems that despite the legislative regulations, consumers consider that improvements are needed. The present work revealed that Eastern European consumers, on average, were not clear about whether the animals that we consume are transported incorrectly (4.4). In that respect, women were a bit (4.5) closer than men to agree with the statement that transportation of animals is inadequate (4.2). Similarly, older people (>55 years old) were more affirmative about that belief than the young ones (<36 years old) Table 2. The observed differences between countries showed that the closest to agree with the statement were Bulgarian consumers (4.8), and the closest to disagree were Hungarian consumers (3.9) Table 3.

The need for improvement was more evident in the case of slaughtering. Thus, when consumers were asked if slaughterhouses should improve slaughter systems to avoid animal suffering, the average score for all Eastern European consumers was above the “Agree” mark (5.4). Women (5.5) agreed more strongly than men (5.2), and consumers that grew up in urban areas (5.5) more strongly than consumers that were raised in rural areas (5.2). Consumers with a mid-class economic status agreed more than those with difficult economic status or those that were wealthy (Table 2). This higher degree of agreement on the need to improve slaughtering may be due to the fact that slaughtering denotes animal’s death and is thus perceived by consumers as more severe for suffering of the animal and, consequently, consumers’ concern [13]. Regarding countries, consumers from Slovenia agreed very strongly and gave significantly highest score (6.7), followed by consumers from Bulgaria (5.7), North Macedonia (5.7), Romania (5.7), and Ukraine (5.6) that agreed strongly. The rest of the investigated countries agreed moderately with the statement, such as Bosnia and Herzegovina, Hungary, and Poland, that gave the lowest scores (4.8–4.9). Citizens are not well informed about the existence of legislation that takes into consideration the way animals are kept on farms, but they know about legislations that considerer transport and slaughter [8]. When asked about animals that are consumed as meat being reared in reduced space, Eastern European consumers agreed that the animals we consume should be reared in adequately sized cages (5.3). The consumers in a difficult economic situation agreed a bit less (5.0) than the middle class and wealthy ones (5.3), and women agreed more (5.4) than men (5.1). Regarding countries (Table 3) only Slovenians agreed strongly (6.2), followed by Czechs, Bulgarians, Croatians, and Romanians (5.5). The lowest agreement to this statement was found for consumers from Bosnia and Herzegovina (4.8) and Poland (4.7), followed by North Macedonia and Moldova (4.9), who responded with average scores that were slightly below the “Agree” mark (5.0).

In addition, regarding the way animals are kept at the farm Eastern European consumer, neither agreed nor disagreed (4.4) with the statement that pigs we consume should be raised in freedom. The score was significantly higher in women than men, in younger consumers (<36 years old), in rural consumers, and in consumers with difficult economic situation. The consumers from Bosnia and Herzegovina (4.9) and Slovenia (4.9) were more inclined to agree with this statement, followed by those from Poland (4.8). The lowest scores to this statement were from consumers from Hungary (4.0), followed by Bulgarians and Romanians (4.1) (Table 3). These results are in a sharp contrast with the results of consumer attitudes surveys conducted in other parts of the Europe, where the animal welfare, among other things, was defined as providing enough space and where extensive and outdoor systems were viewed as more natural and as producing higher quality products [13,14,15]. Canadian citizens also showed a strong preference for animals having access to opened and unenclosed areas, believing that these conditions would result in healthy and happy animals [16].

#### 3.2.2. Beliefs about Ethical Issues about Animals

Eastern European consumers generally agree that the animals that we consume should be treated with dignity (5.4). Women (5.5) agreed slightly, nonetheless statistically significantly more than men (5.3), consumers that grew up in urban (5.5) agree more than in rural (5.3) areas, and consumers with higher education (5.4) more than the ones with only elementary education (5.1). Consumers in a difficult economic situation also agreed but significantly less than consumers from the other economic categories (Table 2). Considering the country, only the consumers from Slovenia agreed strongly with the statement (6.4), while the scores received by the consumers from Bosnia and Herzegovina (4.9) ended up the lowest (Table 3).

Strong disagreement (2.5) with a statement that it does not matter if the animals were mistreated because they will end up as food anyway was observed across the surveyed Eastern European countries. The effect of demographics was not relevant except for the fact that the least pronounced disagreement (2.7) was determined by consumers in a difficult economic situation (Table 2). Slovenian consumers disagreed with this statement very strongly (1.5), which is in accordance with the fact that they also agree strongly with the fact that animals should be treated with dignity. North Macedonian and Polish consumers also disagreed (2.9) Table 3. 

On average, Eastern European consumers were only slightly worried about welfare of animals for human consumption (4.8), but this belief differed according to the country. Bulgarian consumers (3.6) were the least worried about this issue, and Hungarians had no clear opinion (4.1) about it, while the rest of the Eastern European countries exhibited growing concern about welfare of animals for human consumption, with Slovenians being on the upper end of the scale (5.6). This result agrees with the latest Eurobarometer findings about animal welfare [10], where Hungarians and Bulgarians were among the EU countries who believed the least that the welfare of farmed animals should be better protected. The proportion of Eastern European respondents surveyed, who were more or less worried about animal welfare (scores ≥ 5) was 63.1% (data not shown), demonstrating higher concern than observed for U.S. consumers (46%) [4] or lower than observed for the consumers from UK (86%) [17]. In Europe, animal welfare is important for consumers (an average of 7.8 on a scale of 10), spanning between 6.9 and 9.1 depending on the country [9], indicating an important country effect. The socio-demographic analysis in U.S. showed that women were more concerned about animal welfare than men (61% vs 39%, respectively) [4]. The EU survey revealed that women are more likely to “certainly” think that welfare of animals should be better protected than it is now than men (47%, compared to 40%) [10]. Similarly, the present work shows that the concern about welfare of animals for human consumption was slightly, but statistically significantly more expressed, in women (4.9) than men (4.6), but also in consumers with university degree compared to consumers with only elementary education (4.7), and in younger (4.9) than older generations (4.7) (Table 2). The effect of economic status of the consumers was not observed.

#### 3.2.3. Beliefs about Consequences of Animal Welfare on Meat

When asked if taking care of animal welfare produces meat of higher quality, the Eastern European consumers on average agreed with the statement (5.4). This perception was slightly but significantly higher in women than in men, in consumers with urban than rural background, and in consumers that were not in difficult economic situation. The Bulgarians gave the highest average score (5.9), followed by North Macedonians and Slovenians (5.7), whereas Croatians gave the lowest score (4.9), followed by Hungarian, Slovakian, and Bosnian (5.1) consumers (Table 3). The proportion of Eastern European respondents believing that animal welfare has a low impact on better quality of animal products (giving scores ≤ 5) was 84% (data not shown), which is lower than observed for EU residents (EC 2007), who consider buying animal-friendly meat because they consider it healthier (51%) or of better quality (48%). However, better quality is not the dimension that best describes the understanding of animal welfare by EU consumers since only 17% of them considered that animal welfare contributes to better quality animal products [10], with the highest share for Austrians (32%) and Greeks (29%). In contrast to the beliefs of the European consumers, their South African counterparts do not believe that better animal welfare provides better meat quality [18]. On average, Eastern European consumers neither agreed nor disagreed (4.1) with a statement that to ensure animal welfare means eating meat that is more expensive. Minimal but significant effect of education on this matter has been observed because consumers with only elementary school disagreed significantly more (3.7) than consumers with high-school (4.0) or university degrees (4.3). In addition, the agreement with the statement was slightly higher in older consumers (>55 years old) than the younger ones (Table 2). Regarding countries, in general the ‘neither agree nor disagree’ was the most frequent answer. The only exception were consumers from Ukraine (4.9) and Slovenia (4.7), who agreed with such a statement, whereas Bulgarians (3.4), Croatians (3.7), and North Macedonians (3.8) disagreed with it (Table 3). These results are in contrast with the EU public beliefs acknowledging higher production costs in the case of more welfare-friendly farming, i.e., a large majority of EU consumers (72%) support the idea that farmers should be remunerated for the higher costs that can result from greater welfare standards [9].

#### 3.2.4. Willingness to Pay

When Eastern European consumers were asked if they would pay a little more for meat from animals treated with dignity, they overall agreed (4.6). However, they neither agreed nor disagreed with a statement that meat from welfare-friendly animals is more expensive. Consumers that consider themselves to be wealthy and middle class exhibited more willingness to pay (4.7 and 4.6, respectively) than the ones in difficult economic situation (4.3), university degree holders (4.7) more than those with lower education (4.4 and 4.5), younger (<36 years old) (4.7) more than older (>36 years old) (4.5), and women (4.7) more than men (4.4) Table 2.

A similar trend was observed in the recent EU28 investigation [10]. The present results are also in accordance with the previous meta-analysis by Lagerkvist, et al. [19] and Clark, et al. [20]. A survey with Mexican consumers also pointed out that respondents living in cities expressed stronger willingness to pay, as opposed to rural consumers [21]. For EU consumers, it has been shown that willingness to pay more for animal welfare was strictly dependent on the average income [8]. In the present study, the percentage of consumers that gave score 5 or more to this statement was 57.6% (results not shown), which is similar to the EU28 average of 59% [10].

When the differences between countries are considered (Table 3), of all Eastern European consumers, the Slovenians were those willing to pay (5.2) a little more for meat from animals treated with dignity, followed by Moldavians (5.0). The percentage of scores 5 or more in these two countries was 73%. This percentage, in the work from EU28 enquiry [10] was 56% for Slovenia, thus being lower than in the present work. On the other side, Bulgarians gave the lowest score to willingness to pay statement (3.8), followed by Bosnians (4.0) and Hungarians (4.1). In these countries, the percentage of scores 5 or more was 37.8%, 33.6%, and 49.24%, respectively. In the EU28 inquiry [10], the percentage of people that would pay more was 28% in Bulgaria and 40% in Hungary, results slightly higher than those reported in Table 3.

In the EU28 enquiry, consumers that answered positively (they would pay more for welfare-friendly products) versus those that scored 5 or more (the statement willing to pay more for meat from animals treated with dignity) in the present work were 49% versus 60% Croatia, 47% versus 66% in Czech Republic, 41% versus 50% in Slovakia, 40% versus 61% in Romania, and 36% versus 73% in Poland [10]. Furthermore, in the present paper, this percentage was 59% in North Macedonia, 50% in Serbia, and 47% in Ukraine. Other works show that regional differences within Europe, regarding willingness to pay for animal welfare friendly products, are significant [20,22]. Northern Europe countries had lower willingness to pay than the Southern European countries, with UK and North America being in between [20], whereas the data about behavior of Eastern European consumers regarding this matter was never investigated before. The differences among countries in willingness to pay for products from animals treated with dignity show the diversity among the European countries and should influence the European policy decisions because they should ensure meat has an affordable price, despite the extra cost associated with the EU requirements for producing it [20].

### 3.3. Attitudes about Animal Welfare

Overall, Eastern European consumers think that treating animals for human consumption correctly is something good (5.9). Economic situation played a significant role since the consumers considering themselves to be wealthy and middle classed had significantly more positive attitude about it (6.0) than consumers in a difficult economic situation (5.4) Table 4.

Previous studies have not linked increased income with greater concern about farm animal welfare but only with higher willingness to pay [13]. However, in the case of Eastern European consumers, it seems that those with higher incomes might have the means to express their attitudes through their purchasing behaviors, while expressing greater concerns for animal welfare. Regarding countries, the highest scores was observed in Slovakia (6.8), followed by Czech Republic (6.6) and Slovenia (6.4), while consumers in Bosnia and Herzegovina were, more or less, uncertain about it and gave the lowest score (4.7), followed by Polish (5.2) and Serbian consumers (5.3) (Table 5).

Overall, Eastern European consumers were unsure whether treating correctly the animals we consume is easy or difficult (4.3). The effect of demography on this matter was not observed (Table 4). Only those with better economic situation scored a little lower (4.1) than those from mid class and difficult economic situation (4.4). Looking at countries, the highest value (i.e., more difficult) was observed for Slovenian consumers (5.6), followed by Ukrainians (5.3). On the other side, consumers from Czech Republic (3.4) and Bulgaria (2.9) thought the opposite (Table 5).

When consumers were asked if they consider mistreating of the animals we consume as harmful or beneficial, the overall score was 2.1, and small differences were found in demographics. The scores closest to harmful were obtained by consumers from Czech Republic (1.6), Slovenia (1.7), Romania (1.6), and Slovakia (1.6) (Table 5).

The humane treatment of animals for human consumption was overall perceived as natural (2.5), with it being more natural in women (2.4) than in men (2.6), older (2.3) than younger generation (2.7), in urban (2.4) than rural (2.7), and in the middle class (2.4) and wealthy (2.5) consumers more than in those with difficult economic situation (2.9) (Table 4). The closest to score 1 (natural) were for consumers in Czech Republic (1.6). However, consumers from Bosnia and Herzegovina (3.4), Hungary (3.4), Moldova (3.5), and Poland (3.6) thought that treatment of animals for human consumption is neither natural nor artificial (Table 5). The view that livestock has the right to be treated “humanely” was not shared only by the European consumers but also by a vast majority (79–94%) of North American [23] and even Australian (60–71%) consumers [24]. The attitudes of consumers can change over the time; for instance, Chinese consumers today possibly would support changes to improve animal welfare standards [25], while some years ago they were indifferent [26].

### 3.4. Cluster Analysis

Individual consumer preferences can be similar between consumers from different countries. Therefore, a cross-country cluster analysis including all the consumers has been performed to find out segments of consumers with similar beliefs and attitudes. Three clusters of consumers were defined similar in size: cluster 1 (n = 1,734, 32%), cluster 2 (n = 1,674, 31%), and cluster 3 (n = 1,976, 37%). Their characteristics can be found in Table 6 and their beliefs and attitudes in Table 7. Cluster 1 is composed by 56% of Moldavians, 55% of Polish, 54% of Bosnian and Herzegovinian, and 51% of Hungarian consumers (Table 6). No important sociodemographic differences exist between population of this cluster and the general one. It is only composed by slightly lower urban and above 55 years old consumers. Regarding beliefs (Table 7) about particular management aspects, they had quite a neutral opinion in most of them. They are a little concerned about ethical issues about animals since they do not agree in the statement ‘doesn’t matter if we mistreat the animals because we eat them’ (2.9). They are the consumers with the lowest score (4.8) for the statement ‘the animals that we consume should be treated with dignity’. Despite these answers, the rest of their answers were mainly characterized by a high number of respondents positioning themselves in the middle of the scale, indicating that, in general, this group of respondents had quiet neutral scores in most of the beliefs and attitudes, and they could be named “Indifferent towards animal welfare”.

Such answering behavior reflects consumers that express uncertainty and/or unawareness [27], while categorical indifference is a common attitude of respondents towards topics that are not completely comprehended [28]. This group of Eastern European consumers do agree that taking care of animal welfare produces meat of higher quality (5.1), but, at the same time, they were ambiguous about paying a little more for the meat from animals treated with dignity (4.2). Perhaps an obvious explanation lies in the fact that the consumers from Cluster 1 had the largest share of people (38%) living in a difficult economic situation. However, the price is not the only barrier that affects the buying/consuming welfare-friendly products because the lack of trust in claims of the labels and the packaging, the limited availability, the convenience, and the lack of product information may also affect it [29]. One of the limitations of the present study is that this matter has not been explored.

Cluster 2 is composed by 58% of Ukrainians and 51% of Slovenians and only by 11% of Romanian and 12% of Bulgarian (Table 6). Although this group of consumers had the lowest proportion of young (28%) and wealthy (25%) participants, no important differences in the sociodemographic characteristics are seen. This group of consumers agree in the statements that ‘slaughter systems should be improved to avoid animal suffering’ (5.6), ‘avoided rearing animals we consume in much reduced spaces’ (5.4), and ‘the animals that we consume should be treated with dignity’ (5.5) and disagree in the statement ‘doesn’t matter if we mistreat the animals because we eat them’ (2.3) (Table 7). This is in concordance with the fact that they considered that treating animals poorly for human consumption is bad (5.7). Thus, this group of consumers is concerned about animal welfare. Furthermore, they are the only cluster of consumers that considered that correctly treating the animals that we consume is difficult (5.7) and, together with consumers from cluster 3, they considered that humane treatment of animals for human consumption is natural (1.7). Thus, these consumers can be named ‘Consumers concerned about animal welfare, but they believe it is difficult to achieve’. Probably because of that, this is the group of consumers with the highest score in the statement ‘ensuring animal welfare means to eat more expensive meat’ (4.6) and the willingness to pay a little more for meat from animals treated with dignity (4.9), although it is also the group with the lowest proportion of wealthy consumers.

Cluster 3 is mainly composed by Bulgarian (73%) and Czech (64%) consumers and by half of the Romanian ones (50%). It is the cluster with the highest proportion of urban consumers (42%), consumers between 36 and 55 years old (37%), and wealthy consumers (41%). This group of consumers is also concerned about animal welfare since they consider, like cluster 2, that ‘slaughter systems should be improved to avoid animal suffering’ (5.6) and ‘the animals that we consume should be treated with dignity’ (5.7). However, they do not believe that ‘ensuring animal welfare means to eat more expensive meat’ (3.7), and this can explain that this group of consumers considered that correctly treating the animals for human consumption is good (6.4) and easy (2.9), probably because it is also natural (1.6). Thus, this cluster can be named consumers ‘Consumers concerned about animal welfare, and they believe it is possible to achieve it’.

## 4. Conclusions

Survey results are estimations, the accuracy of which, everything being equal, rests upon the sample size and upon the observed percentage. Therefore, a wider survey in order to confirm these preliminary results is needed. Based on the results of the present exploratory study, it is possible to conclude that beliefs and attitudes toward animal welfare differ between consumers from Eastern European countries. The differences among countries show the diversity that exists among them. Some consumers are highly concerned about animal welfare, others are only moderately concerned about animal welfare, and the biggest group of consumers is indifferent towards animal welfare. Considering all these differences, it is important to provide the information to consumers to improve their knowledge about what is going to ensure animal welfare at farm and slaughter plant level, to show them the importance of the humane treatment of animals, and to show the effect of animal treatment on meat quality. Furthermore, the knowledge of the different type of consumers regarding their attitude and beliefs towards animal welfare would help to design campaigns or marketing strategies regarding this topic.

## Figures and Tables

**Table 1:** Demographic profile (%) of the sample of consumers that participate in the study by country ^1^ (*n* = 5384).

	Overall	BIH	BGR	CZE	HRV	MKD	HUN	MDA	POL	ROU	SRB	SVK	SVN	UKR
(%)		(*n* = 309)	(*n* = 352)	(*n* = 506)	(*n* = 301)	(*n* = 284)	(*n* = 400)	(*n* = 299)	(*n* = 504)	(*n* = 556)	(*n* = 661)	(*n* = 296)	(*n* = 215)	(*n* = 701)
Gender														
Male	44.7	51.1	40.9	38.9	39.5	48.2	56	31.1	48.6	49.8	47.7	39.2	46	39.9
Female	55.3	48.9	59.1	61.1	60.5	51.8	44	68.9	51.4	50.2	52.3	60.8	54	60.1
Age														
Less than 36	38.2	23.9	17.6	44.5	33.6	36.3	46	62.2	48.7	45.1	33.4	33.8	47.4	28.7
36–55	34.3	47.3	49.7	25.7	30.6	34.8	37.2	23.4	19.2	30.4	40.3	38.2	34	38.5
Above 55	27.5	28.8	32.7	29.8	35.8	28.9	16.8	14.4	32.1	24.5	26.3	28	18.6	32.8
Educational level														
Elementary	5.4	9.1	4.3	2.4	11.3	7.4	1	3.7	1	10.4	10.3	5.4	5.1	1.1
Higher	42.2	68.6	54	54.9	33.9	53.2	40	45.5	26.2	44.3	62.5	46.6	36.3	5
University	52.4	22.3	41.7	42.7	54.8	39.4	59	50.8	72.8	45.3	27.2	48	58.6	93.9
Place of growing up														
Urban	44.5	85.4	9.4	49.2	56.5	33.5	51	42.5	41.5	42.3	64.9	42.9	72.1	14.4
Rural	55.5	14.6	90.6	50.8	43.5	66.5	49	57.5	58.5	57.7	35.1	57.1	27.9	85.6
Economic situation														
Difficult	21.7	64.7	39.8	12.3	5	3.5	1.3	26.1	13.3	24.8	34.4	4.7	2.3	29.3
Middle class	63.6	34.6	55.1	77.5	88.4	91.9	83.7	65.6	54	30.6	60.8	65.5	79.5	66.3
Wealthy	14.7	0.7	5.1	10.2	6.6	4.6	15	8.3	32.7	44.6	4.8	29.8	18.2	4.4

^1^ BIH: Bosnia and Herzegovina; BGR: Bulgaria; CZE: Czech Republic; HRV: Croatia; MKD: North Macedonia; HUN: Hungary; MDA: Moldova; POL: Poland; ROU: Romania; SRB: Serbia; SVK: Slovakia; SVN: Slovenia; UKR: Ukraine.

**Table 2:** The beliefs of Eastern European consumers about animal welfare and the effect of demographics ^1^.

Beliefs ^4^		Gender		Age			Education ^2^			Growing Place		Economic Situation ^3^		
	Overall	Men	Women	<36	36–55	>55	Elem.	High	Univ.	Urban	Rural	Dif.	Mid.	Wea.
Aspects of management														
The animals that we consume are transported incorrectly	4.4	4.2 ^b^	4.5 ^a^	4.3 ^b^	4.4 ^ab^	4.5 ^a^	4.3	4.4	4.4	4.4 ^a^	4.3 ^b^	4.3	4.4	4.4
^1^ The slaughterhouses should improve the slaughter systems to avoid animals suffering	5.4	5.2 ^b^	5.5 ^a^	5.4	5.4	5.4	5.3 ^b^	5.3 ^b^	5.4 ^a^	5.5 ^a^	5.2 ^b^	5.2 ^b^	5.5 ^a^	5.3 ^a^
It should be avoided rearing animals that we consume in much reduced spaces	5.3	5.1 ^b^	5.4 ^a^	5.3	5.3	5.3	5.2	5.3	5.3	5.3 ^a^	5.2 ^b^	5.0 ^b^	5.3 ^a^	5.3 ^a^
The pigs we consume should grow in freedom	4.4	4.3 ^b^	4.5 ^a^	4.5 ^a^	4.4 ^b^	4.4 ^b^	4.5	4.4	4.4	4.4 ^b^	4.5 ^a^	4.6 ^a^	4.4 ^b^	4.3 ^b^
Ethical issues about animals														
The animals that we consume should be treated with dignity	5.4	5.3 ^b^	5.5 ^a^	5.4	5.4	5.4	5.1 ^b^	5.4 ^ab^	5.4 ^a^	5.5 ^a^	5.3 ^b^	5.2 ^b^	5.5 ^a^	5.4 ^a^
Doesn’t matter if we mistreat the animals because at the end we eat them	2.5	2.5	2.5	2.4	2.5	2.5	2.5 ^a^	2.4 ^b^	2.5 ^ab^	2.4	2.5	2.7 ^a^	2.4 ^b^	2.5 ^b^
I am worried about welfare of animals for human consumption	4.8	4.6 ^b^	4.9 ^a^	4.9 ^a^	4.7 ^b^	4.7 ^b^	4.7 ^b^	4.6 ^b^	4.9 ^a^	4.8	4.8	4.7	4.7	4.8
Consequences of animal welfare on meat														
Taking care of animal welfare produces meat of higher quality	5.4	5.3 ^b^	5.4 ^a^	5.4	5.3	5.4	5.3	5.4	5.4	5.4 ^a^	5.3 ^b^	5.3 ^b^	5.4 ^a^	5.4 ^a^
To ensure animal welfare means to eat meat that is more expensive	4.1	4.1	4.2	4.1 ^b^	4.1 ^b^	4.3 ^a^	3.7 ^b^	4.0 ^a^	4.3 ^a^	4.2	4.1	4.2	4.1	4.1
Willingness to pay														
I am willing to pay a little more for meat from animals treated with dignity	4.6	4.4 ^b^	4.7 ^a^	4.7 ^a^	4.5 ^b^	4.5 ^b^	4.4 ^b^	4.5 ^b^	4.7 ^a^	4.6 ^a^	4.5 ^b^	4.3 ^b^	4.6 ^a^	4.7 ^a^

^1. a,b^ in the same row and within the same socio-demographic variable with different letters are significantly different at the level of 5%. ^2^ Elementary, High school, University; ^3^ Difficult, Mid class, Wealthy; ^4^ (1)-Disagree very strongly; (2)-Disagree strongly; (3)-Disagree; (4)-Neither agree nor disagree; (5)-Agree; (6)-Agree strongly; (7)-Agree very strongly.

**Table 3:** The beliefs of Eastern European consumers about animal welfare and the effect of the country ^1^.

Beliefs ^4^	BIH ^2^	BGR	CZE	HRV	MKD	HUN	MDA	POL	ROU	SRB	SVK	SVN	UKR	RMSE ^3^
Aspects of management														
The animals that we consume are transported incorrectly	4.5 ^ab^	4.8 ^a^	4.4 ^bc^	4.6 ^ab^	4.1 ^cd^	3.9 ^d^	4.6 ^ab^	4.5 ^ab^	4.3 ^bc^	4.3 ^bc^	4.4 ^bc^	4.5 ^ab^	4.4 ^bc^	1.4
The slaughterhouses should improve the slaughter systems to avoid animal suffering	4.8 ^e^	5.7 ^b^	5.3 ^cd^	5.5 ^bcd^	5.7 ^b^	4.9 ^e^	5.3 ^cd^	4.9 ^e^	5.7 ^b^	5.3 ^cd^	5.2 ^d^	6.7 ^a^	5.6 ^bc^	1.4
It should be avoided rearing animals that we consume in much reduced spaces	4.8 ^e^	5.5 ^b^	5.5 ^b^	5.5 ^b^	4.9 ^de^	5.4 ^bc^	4.9 ^de^	4.7 ^e^	5.5 ^b^	5.4 ^bc^	5.1 ^cd^	6.2 ^a^	5.3 ^bc^	1.4
The pigs we consume should grow in freedom	4.9 ^a^	4.1 ^de^	4.5 ^bc^	4.4 ^cd^	4.2 ^cde^	4.0 ^e^	4.6 ^abc^	4.8 ^ab^	4.1 ^de^	4.4 ^cd^	4.6 ^abc^	4.9 ^a^	4.4 ^cde^	1.6
Ethical issues about animals														
Animal we consume should be treated with dignity	4.9 ^h^	5.9 ^b^	5.7 ^bc^	5.3 ^def^	5.6 ^bcd^	5.0 ^gh^	5.3 ^efg^	5.1 ^fgh^	5.6 ^bcd^	5.2 ^efg^	5.2 ^efgh^	6.4 ^a^	5.4 ^cde^	1.3
Does not matter if we mistreat the animals because at the end we eat them	2.5 ^bcd^	2.3 ^cde^	2.1 ^e^	2.6 ^ab^	2.1 ^e^	2.0 ^e^	2.9 ^a^	2.9 ^a^	2.2 ^de^	2.6 ^abc^	2.7 ^ab^	1.5 ^f^	2.8 ^ab^	1.4
I am worried about welfare of animals for human consumption	4.8 ^cde^	3.6 ^h^	4.5 ^ef^	4.5 ^def^	4.9 ^cd^	4.1 ^g^	5.1 ^bc^	4.7 ^def^	5.2 ^b^	4.9 ^cd^	4.4 ^f^	5.6 ^a^	5.2 ^b^	1.5
Consequences of animal welfare on meat														
Taking care of animal welfare produces meat of higher quality	5.1 ^ef^	5.9 ^a^	5.5 ^bcd^	4.9 ^f^	5.7 ^ab^	5.1 ^ef^	5.5 ^abcd^	5.1 ^ef^	5.6 ^abc^	5.3 ^de^	5.1 ^ef^	5.7 ^ab^	5.4 ^cde^	1.3
To ensure animal welfare means to eat meat that is more expensive	4.4 ^bc^	3.4 ^f^	4.0 ^cde^	3.7 ^e^	3.8 ^e^	4.3 ^bc^	4.2 ^c^	4.2 ^cd^	3.8 ^de^	4.3 ^c^	3.7 ^ef^	4.7 ^ab^	4.9 ^a^	1.5
Willingness to pay														
I am willing to pay a little more for meat from animals treated with dignity	4.0 ^fg^	3.8 ^g^	4.8 ^bcd^	4.7 ^bcd^	4.6 ^cde^	4.1 ^fg^	5.0 ^ab^	4.7 ^bcd^	4.9 ^abc^	4.5 ^de^	4.3 ^ef^	5.2 ^a^	4.9 ^bcd^	1.5

^1 a–e^ Items in the same row with different letters are significantly different at the level of 5%.; ^2^ BIH: Bosnia and Herzegovina; BGR: Bulgaria; CZE: Czech Republic; HRV: Croatia; MKD: North Macedonia; HUN: Hungary; MDA: Moldova; POL: Poland; ROU: Romania; SRB: Serbia; SVK: Slovakia; SVN: Slovenia; UKR: Ukraine. ^3^ RMSE: root-mean-square error; ^4^ (1)-Disagree very strongly; (2)-Disagree strongly; (3)-Disagree; (4)-Neither agree nor disagree; (5)-Agree; (6)-Agree strongly; (7)-Agree very strongly.

**Table 4:** The attitudes of Eastern European consumers towards castration and the effect of demographics ^1^.

		Gender		Age			Education ^2^			Growing Place		Economic Situation ^3^		
	Overall	Men	Women	<36	36–55	>55	Elem.	High	Univ.	Urban	Rural	Dif.	Mid.	Wea.
Treating correctly animals for human consumption is …	5.9	5.8 ^b^	5.9 ^a^	5.9 ^c^	5.7 ^b^	6.0 ^a^	5.7 ^b^	5.9 ^a^	5.9 ^a^	6.0 ^b^	5.7 ^a^	5.4 ^b^	6.0 ^a^	6.0 ^a^
(1)-Bad; (4)-Neither bad nor good; (7)-Good														
Treating correctly the animals that we consume is…	4.3	4.3	4.3	4.3	4.3	4.3	4.1	4.2	4.5	4.3	4.4	4.4 ^a^	4.4 ^a^	4.1 ^b^
(1)-Easy; (4)-Neither easy nor difficult; (7)-Difficult														
To mistreat the animals we consume is …	2.1	2.2	2.1	2.1 ^b^	2.2 ^a^	2.1 ^b^	2.2	2.1	2.1	2.2	2.1	2.2 ^a^	2.0 ^b^	2.1 ^ab^
(1)-Harmful; (4)-Neither harmful nor beneficial; (7)-Beneficial														
Humane treatment of animals for human consumption is …	2.5	2.6 ^a^	2.4 ^b^	2.7 ^a^	2.5 ^b^	2.3 ^c^	2.4 ^b^	2.6 ^a^	2.4 ^b^	2.4 ^b^	2.7 ^a^	2.9 ^a^	2.4 ^b^	2.5 ^b^
(1)-Natural; (4)-Neither natural nor artificial; (7)-Artificial														

^1 a,b^ Items in the same row and within the same socio-demographic variable with different letters are significantly different at the level of 5%. ^2^ Elementary, High school, University; ^3^ Difficult, Mid class, Wealthy.

**Table 5:** The attitudes of Eastern European consumers about castration and the effect of the country ^1^.

	^2^ BIH	BGR	CZE	HRV	MKD	HUN	MDA	POL	ROU	SRB	SVK	SVN	UKR	RMSE ^3^
Treating correctly animals for human consumption is … (1)-Bad; (4)-Neither bad nor good; (7)-Good	4.7 ^f^	5.8 ^d^	6.6 ^ab^	6.0 ^cd^	6.1 ^cd^	5.2 ^e^	6.0 ^cd^	5.2 ^e^	6.2 ^c^	5.3 ^e^	6.8 ^a^	6.4 ^bc^	6.2 ^c^	1.5
In my opinion treating correctly the animals that we consume is … (1)-Easy; (4)-Neither easy nor difficult; (7)-Difficult	4.9 ^cd^	2.9 ^g^	3.4 ^f^	4.1 ^e^	4.5 ^de^	5.0 ^bc^	4.6 ^de^	4.3 ^e^	3.4 ^f^	4.1 ^e^	4.9 ^bcd^	5.6 ^a^	5.3 ^ab^	2.0
To mistreat the animals we consume is …	2.5 ^ab^	2.4 ^bc^	1.6 ^f^	2.4 ^bc^	2.0 ^cde^	1.9 ^def^	2.2 ^bcd^	2.8 ^a^	1.6 ^f^	2.4 ^b^	1.6 ^ef^	1.7 ^ef^	2.3 ^bc^	1.8
(1)-Harmful; (4)-Neither harm nor beneficial; (7)-Beneficial														
I think that humane treatment of animals for human consumption is …	3.4 ^a^	2.6 ^b^	1.6 ^e^	2.0 ^cd^	2.1 ^c^	3.4 ^a^	3.5 ^a^	3.6 ^a^	2.8 ^b^	2.6 ^b^	1.6 ^de^	1.9 ^cde^	1.8 ^cde^	1.8
(1)-Natural; (4)-Neither natural nor artificial; (7)-Artificial														

^1 a–f^ Items in the same row denoted with different letters are significantly different at the level of 5%. ^2^ BIH: Bosnia and Herzegovina; BGR: Bulgaria; CZE: Czech Republic; HRV: Croatia; MKD: North Macedonia; HUN: Hungary; MDA: Moldova; POL: Poland; ROU: Romania; SRB: Serbia; SVK: Slovakia; SVN: Slovenia; UKR: Ukraine. ^3^ RMSE: root-mean-square error.

**Table 6:** Description of the three clusters in terms of demographics.

%	Cluster 1 (*n* = 1734)	Cluster 2 (*n* = 1674)	Cluster 3 (*n* = 1976)	Total (*n* = 5384)
Country				
Bosnia and Herzegovina	54.2	35.7	10.0	309
Bulgaria	14.5	12.2	73.3	352
Czech Republic	14.4	21.7	63.8	506
Croatia	25.3	35.2	39.5	301
North Macedonia	22.8	36.1	41.1	284
Hungary	50.5	32.3	17.3	400
Moldova	56.0	23.0	21.0	299
Poland	55.4	25.0	19.6	504
Romania	38.1	11.7	50.2	556
Serbia	37.6	27.2	35.2	661
Slovakia	18.2	39.9	41.9	296
Slovenia	20.4	51.1	28.5	215
Ukraine	13.3	57.8	29.0	701
Gender				
Male	35.0	28.2	36.9	2404
Female	30.0	33.5	36.5	2980
Growing place				
Urban	26.0	32.3	41.7	2986
Rural	36.4	30.3	33.3	2398
Age				
Less than 36	36.3	27.8	36.0	2055
36–55	32.0	30.7	37.4	1849
Above 55	26.8	36.4	36.8	1480
Economic situation				
Difficult	38.3	34.1	27.7	1166
Middle class	29.7	31.5	38.8	3425
Wealthy	34.2	25.0	40.9	793

**Table 7:** Description of the three clusters regarding beliefs, willingness to pay, and attitudes toward castration and meat from castrated pigs ^1^.

	Cluster 1 (*n* = 1734)	Cluster 2 (*n* = 1674)	Cluster 3 (*n* = 1976)	RMSE ^3^
Beliefs about particular aspects of management ^2^				
The animals that we consume are transported incorrectly	4.2 ^b^	4.5 ^a^	4.5 ^a^	1.4
Slaughter systems should be improved to avoid animal suffering	4.9 ^b^	5.6 ^a^	5.6 ^a^	1.4
Avoided rearing animals we consume in much reduced spaces	4.9 ^b^	5.4 ^a^	5.5 ^a^	1.4
The pigs we consume should grow in freedom	4.2 ^b^	4.8 ^a^	4.3 ^b^	1.6
Beliefs about ethical issues about animals ^2^				
The animals that we consume should be treated with dignity	4.8 ^c^	5.5 ^b^	5.7 ^a^	1.2
Doesn’t matter if we mistreat the animals because we eat them	2.9 ^a^	2.3 ^b^	2.3 ^b^	1.4
I am worried about welfare of animals for human consumption	4.6 ^b^	4.9 ^a^	4.8 ^a^	1.5
Beliefs about consequences of animal welfare on meat ^2^				
Taking care of animal welfare produces meat of higher quality	5.1 ^c^	5.3 ^b^	5.6 ^a^	1.3
Ensuring animal welfare means to eat more expensive meat	4.2 ^b^	4.6 ^a^	3.7 ^c^	1.5
Willingness to pay ^2^				
Willing to pay a little more for meat from animals treated with dignity	4.2 ^c^	4.9 ^a^	4.6 ^b^	1.5
Attitude towards animal welfare				
Treating correctly animals for human consumption is …	5.4 ^c^	5.7 ^b^	6.4 ^a^	1.5
(1)-Bad; (4)-Neither bad nor good; (7)-Good				
Treating correctly the animals that we consume is…	4.6 ^b^	5.7 ^a^	2.9 ^c^	1.6
(1)-Easy; (4)-Neither easy nor difficult; (7)-Difficult				
To mistreat the animals we consume is …	2.4 ^a^	2.4 ^a^	1.5 ^b^	1.4
(1)-Harmful; (4)-Neither harmful nor beneficial; (7)-Beneficial				
Humane treatment of animals for human consumption is …	4.5 ^a^	1.7 ^b^	1.6 ^b^	1.3
(1)-Natural; (4)-Neither natural nor artificial; (7)-Artificial				

^1 a,b^ Same row denoted with different superscript are significantly different at the level of 5%; ^2^ (1)-Disagree very strongly; (4)-Neither agree nor disagree; (7)-Agree very strongly. ^3^ RMSE: root mean square error.

## Supplementary Materials

The following are available online at https://www.mdpi.com/2076-2615/10/7/1220/s1, Table S1(X): Description of EU/non-EU countries regarding beliefs, willingness to pay and attitudes toward castration and meat from castrated pigs1.
