# Supplementary material for: Attitudes and Beliefs of Eastern European Consumers Towards Animal Welfare
Source: Animals (Basel). 2020 Jul 17;10(7):1220. doi: 10.3390/ani10071220 (PMC7401545; doi:10.3390/ani10071220)
Supplement: Supplementary file 1 [file animals-10-01220-s001.pdf]

Table X: Description of EU / non-EU countries regarding beliefs, willingness to pay and attitudes toward castration and meat from castrated pigs[1].

	EU (n=3,130)	Non-EU (n=2,254)
Beliefs about particular aspects of management[2]		
The animals that we consume are transported incorrectly	4.4	4.4
Slaughter systems should be improved to avoid animal suffering	5.4	5.4
Avoided rearing animals we consume in much reduced spaces	**5.4**[a]	**5.2**[b]
The pigs we consume should grow in freedom	4.4	4.5
Beliefs about ethical issues about animals[2]		
The animals that we consume should be treated with dignity	**5.5**[a]	**5.3**[b]
Doesn't matter if we mistreat the animals because we eat them	2.4	2.6
I am worried about welfare of animals for human consumption	**4.6**[a]	**5.0**[b]
Beliefs about consequences of animal welfare on meat[2]		
Taking care of animal welfare produces meat of higher quality	5.4	5.4
Ensuring animal welfare means to eat more expensive meat	**4.0**[a]	**4.4**[b]
Willingness to pay[2]		
Willing to pay a little more for meat from animals treated with dignity	4.6	4.6
Attitude towards animal welfare		
Treating correctly animals for human consumption is …	**6.0**[a]	**5.7**[b]
(1) - Bad; (4) - Neither bad nor good; (7) - Good		
Treating correctly the animals that we consume is…	4.2	4.5
(1) - Easy; (4) - Neither easy nor difficult; (7) - Difficult		
To mistreat the animals we consume is …	2.3	2.1
(1) - Harmful; (4) - Neither harmful nor beneficial; (7) - Beneficial		
Humane treatment of animals for human consumption is …	2.5	2.5
(1) - Natural; (4) - Neither natural nor artificial; (7) - Artificial		

[1] Same row denoted with different superscript are significantly different at the level of 5%; [2]*(1) - Disagree very strongly; (4) Neither agree nor disagree; (7) - Agree very strongly.*

EU (Bulgaria, Czech Republic, Croatia, Hungary, Poland, Romania, Slovakia, Slovenia); Non-EU (Bosnia and Herzegovina, North Macedonia, Moldova, Serbia, Ukraine)
